# Altering the mechanical scenario to decrease the driving pressure

**DOI:** 10.1186/s13054-015-1063-x

**Published:** 2015-09-21

**Authors:** João Batista Borges, Göran Hedenstierna, Anders Larsson, Fernando Suarez-Sipmann

**Affiliations:** Hedenstierna Laboratory, Department of Surgical Sciences, Section of Anaesthesiology & Critical Care, Uppsala University, Hospital, 75185 Uppsala, Sweden; Hedenstierna Laboratory, Department of Medical Sciences, Clinical Physiology, Uppsala University, Hospital, 75185 Uppsala, Sweden

## Abstract

Ventilator settings resulting in decreased driving pressure (ΔP) are positively associated with survival. How to further foster the potential beneficial mediator effect of a reduced ΔP? One possibility is promoting the active modification of the lung’s “mechanical scenario” by means of lung recruitment and positive end-expiratory pressure selection. By taking into account the individual distribution of the threshold-opening airway pressures to achieve maximal recruitment, a redistribution of the tidal volume from overdistended to newly recruited lung occurs. The resulting more homogeneous distribution of transpulmonary pressures may induce a relief of overdistension in the upper regions. The gain in lung compliance after a successful recruitment rescales the size of the functional lung, potentially allowing for a further reduction in ΔP.

Amato et al. [1] showed that ventilator settings resulting in decreased driving pressure (ΔP) were positively associated with survival. One may reason that ΔP scales the tidal volume in relation to the aerated lung size and the mechanical scenario created by the positive end-expiratory pressure (PEEP) being used, implying that a lung-protective ventilatory strategy may be adapted to the size of the aerated lung. The same was concluded based on the association of tidal hyperinflation with higher concentrations of inflammatory mediators and a lower number of ventilator-free days in acute respiratory distress syndrome patients who had larger amounts of collapse despite tidal volume and pressure limitations [2]. Experimental data emphasize the importance of unloading the small-aerated lung [3], which receives most of ventilation, because strain hotspots concentrate as much as four times more in alveolar walls of aerated units that are overstretched.

How to further foster the potential beneficial mediator effect of a reduced ΔP? We think that an attractive alternative possibility could consist of promoting the active modification of the lung’s “mechanical scenario” by means of lung recruitment and careful PEEP selection (Fig. 1). By stepwise challenging the lung, taking into account the individual distribution of the threshold-opening airway pressures to achieve maximal recruitment, a redistribution of the tidal volume from overdistended to newly recruited lung occurs. The resulting more homogeneous distribution of transpulmonary pressures through interdependence properties may induce a relief of overdistension in the upper regions [4]. In addition, the gain in lung compliance after a successful recruitment rescales the size of the functional lung, potentially allowing for a further reduction in ΔP without decreasing ventilation.

**Fig. 1:**
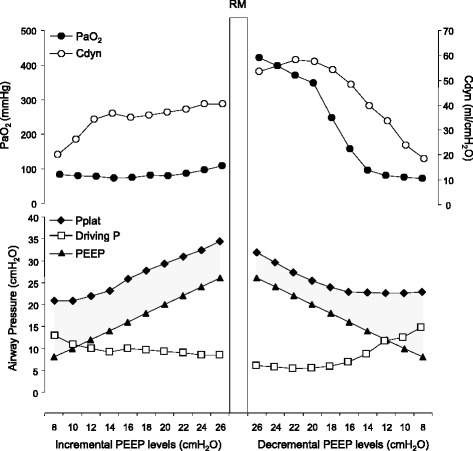
Representative data for a patient with early pulmonary acute respiratory distress syndrome (pneumonia) submitted to incremental positive end-expiratory pressure (*PEEP*) levels followed by an individualized recruitment maneuver (*RM*) and a subsequent decremental PEEP titration. The same PEEP levels were applied during the incremental and decremental phases. The patient was ventilated in a volume-controlled mode with a tidal volume of 6 ml/kg (predicted body weight). During the incremental phase, dynamic compliance (*Cdyn*) responded with an initial increase until PEEP 14 cmH_2_O and then remained constant with further incremental steps without any effects on oxygenation. This indicated a lack of any relevant recruitment effect during the incremental steps, which resulted in a constant driving pressure (*Driving P*). A new mechanical scenario was achieved after lung recruitment, confirmed by the increase in compliance and oxygenation already at the first decremental PEEP steps. As a consequence, ΔP decreased (*lower panel*, *shaded area*) despite ventilation at a similar tidal volume. The decreased ΔP was maintained until the beginning of lung collapse during the decremental PEEP steps, evidenced by the fall in oxygenation and compliance. This example illustrates how the modulatory effects of ΔP can be optimized when the mechanical scenario is modified while minimizing lung collapse by recruitment and PEEP titration. Appropriate ethics approval was obtained, as well as appropriate consents to publish from the participant (or legal parent or guardian for children) to report individual patient data. *PaO*_*2*_ arterial partial pressure of oxygen, *Pplat* plateau pressure

Lung recruitment is a complex process whose effects depend on the amount of recruitment achieved and the level of PEEP necessary to stabilize the lung. A recurrent concern regarding recruitment maneuvers is overdistension. We stress the counterintuitive and opposite effects of partial/nonindividualized versus maximum/titrated recruitment strategies [4]. A partial recruitment may exacerbate local stretches because the persistence of lung collapse maintains the heterogeneous distribution of ventilation [2]. Any recruitment strategy should therefore aim at the individual maximal possible recruitment to obtain its highest benefit.

Under the “newly created” circumstances of a fully recruited lung, it is essential to determine and maintain an end-expiratory pressure level above the closing pressure of the newly recruited lung and use the minimum possible ΔP. This not only sustains the improved mechanical scenario of a resized more homogeneous lung, but also attenuates the derecruitment-associated lung injury [4].

## Abbreviations

PEEP: Positive end-expiratory pressure
ΔP: Driving pressure
